# Supplementary material for: Racial discrimination, low trust in the health system and COVID-19 vaccine uptake: a longitudinal observational study of 633 UK adults from ethnic minority groups
Source: J R Soc Med. 2022 May 5;115(11):439–47. doi: 10.1177/01410768221095241 (PMC9723809; doi:10.1177/01410768221095241)
Supplement: sj-pdf-1-jrs-10.1177_01410768221095241 - Supplemental material for Racial discrimination, low trust in the health system and COVID-19 vaccine uptake: a longitudinal observational study of 633 UK adults from ethnic minority groups [file sj-pdf-1-jrs-10.1177_01410768221095241.pdf]

Supplemental Materials

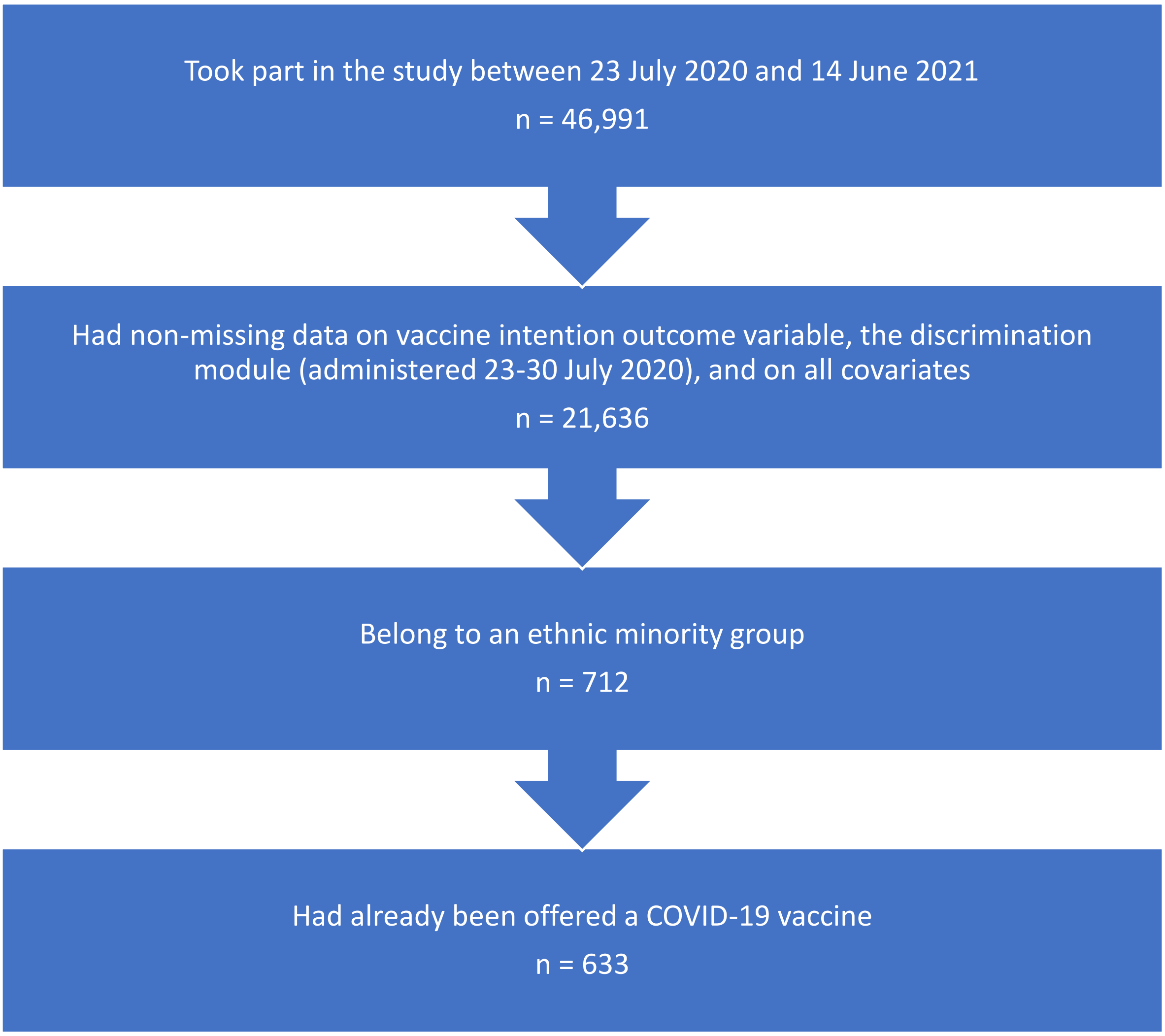

Figure S1. Flow chart of sample selection

Table S1. Characteristics of included and excluded participants, unweighted

Variable	Excluded N = 46,358 %	Included N = 633 %
Ethnicity		
Asian/Asian British - Indian, Pakistani, Bangladeshi, other	30.36	27.49
Black/Black British - Caribbean, Africa	11.69	13.43
Mixed race - White and Black/Black British	10.60	8.21
Mixed race - other	25.18	27.96
Chinese/Chinese British	7.71	6.32
Middle Eastern/Middle Eastern British – Arab, Turkish, other	3.37	2.53
Other ethnic group	11.08	14.06
Gender (ref male)		
Female	79.52	76.30
Education (ref university degree or higher)		
A-levels/equivalent or vocational	13.73	10.74
Up to GCSE/ O levels	7.83	8.53
Age (ref 60 +)		
45-59	31.93	37.76
30-44	38.55	28.59
18-29	16.87	5.53
Long-term physical health condition (ref do not have)		
Have a long-term physical health condition	35.18	46.29
COVID-19 infection status (ref not been infected)		
Have been infected w/COVID-19	3.98	2.53
Catching or becoming seriously ill from COVID-19 a source of stress		
Not a source of stress	71.57	66.98
Low confidence in the central UK government (ref high)		
Low	64.22	69.19
Low confidence in the UK health service (ref high)		
Low	27.23	23.70
Racial/ethnic discrimination experiences		
You have been treated with less courtesy or respect than other people	12.50	11.22
You have received poorer service than other people (e.g., for deliveries or in stores).	5.21	3.79
People have acted as if they were afraid of you	7.29	7.11
People have acted as if they think you are dishonest	2.08	3.48
You have been threatened or harassed	5.21	2.21
You have received poorer service or treatment than other people from doctors or hospitals	0.00	1.11
You have experienced some other kind of discrimination	7.29	6.48
Racial/ethnic discrimination total, M (SD)	0.40 (0.89)	0.35 (0.92)

Table S2. Wording of study-developed and modified items

Variable	Question wording	Response options	Dates of collection
Confidence in government to handle pandemic	To what extent do you have confidence in the central UK Government that they can handle COVID-19 well? [Please answer this question about the government in Westminster, even if you live in a devolved nation]	1. None at all<br>7. Lots	21 March 2020-
Confidence in health system to handle pandemic	How much confidence do you have that the UK health service can cope during COVID-19? If you live in a devolved nation, we ask you to focus on the health service within your country (e.g., NHS Health Scotland / NHS Wales / HSCNI)?	1. None at all<br>7. Lots	21 March 2020-
Racial discrimination	Since lockdown came in, have any of the following things happened to you?<br>1. You have been treated with less courtesy or respect than other people.<br>2. You have received poorer service than other people (e.g., for deliveries or in stores).<br>3. People have acted as if they were afraid of you<br>4. People have acted as if they think you are dishonest<br>5. You have been threatened or harassed<br>6. You have received poorer service or treatment than other people from doctors or hospitals<br>7. You have experienced some other kind of discrimination	1. No<br>2. Yes, because of my gender<br>3. Yes, because of my race/ethnicity<br>4. Yes, because of my age<br>5. Yes, for another reason	23 to 30 July 2020
Not stressed about catching or becoming seriously ill from COVID-19	Over the past week, have any of the following been worrying you at all, even if only in a minor way?<br>Have any of these things been causing you SIGNIFICANT stress? (e.g., they have been constantly on your mind or have been keeping you awake at night)	Catching COVID-19<br>Becoming seriously ill from COVID-19	21 March 2020-
	Have you ever been offered a vaccine for COVID-19?	1. Yes, I have been vaccinated twice<br>2. Yes, I have been vaccinated once<br>3. Yes, but I am waiting to be vaccinated<br>4. Yes, but I have turned it down<br>5. No, I have not yet been offered a vaccine for COVID-19	8 January 2021-
COVID-19 vaccination status	How likely to do you think you are to get a COVID-19 vaccine when one is approved?	1. Very unlikely<br>6. Very likely<br>7. I have already had one	23 December 2020 - 25 February 2021

Table S3. Sensitivity analysis: Direct effects of age discrimination, confidence in government and the health system and COVID-19 vaccine refusal from the structural equation model (N = 633)

	Low confidence in the central UK government			Low confidence in the UK health system			COVID-19 vaccine refusal		
	OR	95% CI	95% CI	OR	95% CI	95% CI	OR	95% CI	95% CI
Low confidence in the central UK government (ref high)	-	-	-	-	-	-	1.10	0.19	6.49
Low confidence in the UK health service (ref high)	-	-	-	-	-	-	**7.68**	**2.13**	**27.74**
Age discrimination	1.49	0.72	3.10	**2.07**	**1.28**	**3.33**	0.81	0.30	2.21
Model fit									
Akaike's information criteria (AIC)					2726.40				
Schwarz's Bayesian information criteria (BIC)					2882.16				
Log pseudolikelihood					-1328.20				

Note. Bold indicates $p < 0.05$. Data were weighted to the proportions of gender, age, ethnicity, country, and education obtained from the Office for National Statistics. Structural equation model adjusts for COVID-19 infection status, not being worried about catching or becoming seriously ill from COVID-19, gender, age, education level, and the presence of a long-term physical health condition.

Table S4. Sensitivity analysis: Indirect effects of age discrimination, confidence in government and the health system and COVID-19 vaccine refusal from the structural equation model (N = 633)

	COVID-19 vaccine refusal		
	OR	95% CI	95% CI
Indirect effect of age discrimination on COVID-19 vaccine refusal through low confidence in the central UK government	1.04	0.51	2.11
Indirect effect of age discrimination on COVID-19 vaccine refusal through low confidence in the UK health service	**4.41**	**1.16**	**16.74**
Total effect of age discrimination on COVID-19 vaccine refusal (direct effect of age discrimination plus indirect effects of low confidence in government and the health system)	3.73	0.71	19.65

Note. Bold indicates $p < 0.05$. Data were weighted to the proportions of gender, age, ethnicity, country, and education obtained from the Office for National Statistics. Structural equation model adjusts for COVID-19 infection status, not being worried about catching or becoming seriously ill from COVID-19, gender, age, education level, and the presence of a long-term physical health condition.

Table S5. Sensitivity analysis: Direct effects of gender discrimination, confidence in government and the health system and COVID-19 vaccine refusal from the structural equation model (N = 633)

	Low confidence in the central UK government			Low confidence in the UK health system			COVID-19 vaccine refusal		
	OR	95% CI	95% CI	OR	95% CI	95% CI	OR	95% CI	95% CI
Low confidence in the central UK government (ref high)	-	-	-	-	-	-	1.07	0.18	6.42
Low confidence in the UK health service (ref high)	-	-	-	-	-	-	**6.89**	**1.96**	**24.16**
Gender discrimination	1.85	0.83	4.15	**2.84**	**1.43**	**5.65**	1.61	0.90	2.89
Model fit									
Akaike's information criteria (AIC)				2715.932					
Schwarz's Bayesian information criteria (BIC)				2871.699					
Log pseudolikelihood				-1322.97					

Note. Bold indicates $p < 0.05$. Data were weighted to the proportions of gender, age, ethnicity, country, and education obtained from the Office for National Statistics. Structural equation model adjusts for COVID-19 infection status, not being worried about catching or becoming seriously ill from COVID-19, gender, age, education level, and the presence of a long-term physical health condition.

Table S6. Sensitivity analysis: Indirect effects of gender discrimination, confidence in government and the health system and COVID-19 vaccine refusal from the structural equation model (N = 633)

	COVID-19 vaccine refusal		
	OR	95% CI	95% CI
Indirect effect of gender discrimination on COVID-19 vaccine refusal through low confidence in the central UK government	1.04	0.35	2.15
Indirect effect of gender discrimination on COVID-19 vaccine refusal through low confidence in the UK health service	**7.49**	**1.11**	**50.50**
Total effect of gender discrimination on COVID-19 vaccine refusal (direct effect of gender discrimination plus indirect effects of low confidence in government and the health system)	**12.57**	**1.37**	**115.58**

Note. Bold indicates $p < 0.05$. Data were weighted to the proportions of gender, age, ethnicity, country, and education obtained from the Office for National Statistics. Structural equation model adjusts for COVID-19 infection status, not being worried about catching or becoming seriously ill from COVID-19, gender, age, education level, and the presence of a long-term physical health condition.

Table S7. Sensitivity analysis: Direct effects of other discrimination, confidence in government and the health system and COVID-19 vaccine refusal from the structural equation model (N = 633)

	Low confidence in the central UK government			Low confidence in the UK health system			COVID-19 vaccine refusal		
	OR	95% CI	95% CI	OR	95% CI	95% CI	OR	95% CI	95% CI
Low confidence in the central UK government (ref high)	-	-	-	-	-	-	1.08	0.18	6.69
Low confidence in the UK health service (ref high)	-	-	-	-	-	-	**6.76**	**1.89**	**24.18**
Other discrimination	1.27	0.98	1.64	1.18	0.87	1.60	**1.59**	**1.11**	**2.26**
Model fit									
Akaike's information criteria (AIC)					2737.81				
Schwarz's Bayesian information criteria (BIC)					2893.57				
Log pseudolikelihood					-1333.90				

Note. Bold indicates $p < 0.05$. Data were weighted to the proportions of gender, age, ethnicity, country, and education obtained from the Office for National Statistics. Structural equation model adjusts for COVID-19 infection status, not being worried about catching or becoming seriously ill from COVID-19, gender, age, education level, and the presence of a long-term physical health condition.

Table S8. Sensitivity analysis: Indirect effects of other discrimination, confidence in government and the health system and COVID-19 vaccine refusal from the structural equation model (N = 633)

	COVID-19 vaccine refusal		
	OR	95% CI	95% CI
Indirect effect of other discrimination on COVID-19 vaccine refusal through low confidence in the central UK government	1.01	0.75	1.36
Indirect effect of other discrimination on COVID-19 vaccine refusal through low confidence in the UK health service	1.58	0.86	2.90
Total effect of other discrimination on COVID-19 vaccine refusal (direct effect of other discrimination plus indirect effects of low confidence in government and the health system)	**2.54**	**1.09**	**5.89**

Note. Bold indicates $p < 0.05$. Data were weighted to the proportions of gender, age, ethnicity, country, and education obtained from the Office for National Statistics. Structural equation model adjusts for COVID-19 infection status, not being worried about catching or becoming seriously ill from COVID-19, gender, age, education level, and the presence of a long-term physical health condition.

Table S9. Sensitivity analysis: Direct effects of discrimination in medical settings, confidence in government and the health system and COVID-19 vaccine refusal from the structural equation model (N = 633)

	Low confidence in the central UK government			Low confidence in the UK health system			COVID-19 vaccine refusal		
	OR	95% CI	95% CI	OR	95% CI	95% CI	OR	95% CI	95% CI
Low confidence in the central UK government (ref high)	-	-	-	-	-	-	1.11	0.19	6.57
Low confidence in the UK health service (ref high)	-	-	-	-	-	-	**8.06**	**2.22**	**29.22**
Discrimination in medical settings	0.55	0.21	1.44	0.91	0.33	2.45	3.05	0.90	10.27
Model fit									
Akaike's information criteria (AIC)					2748.78				
Schwarz's Bayesian information criteria (BIC)					2904.55				
Log pseudolikelihood					-1339.39				

Note. Bold indicates $p < 0.05$. Data were weighted to the proportions of gender, age, ethnicity, country, and education obtained from the Office for National Statistics. Structural equation model adjusts for COVID-19 infection status, not being worried about catching or becoming seriously ill from COVID-19, gender, age, education level, and the presence of a long-term physical health condition.

Table S10. Sensitivity analysis: Indirect effects of discrimination in medical settings, confidence in government and the health system and COVID-19 vaccine refusal from the structural equation model (N = 633)

	COVID-19 vaccine refusal		
	OR	95% CI	95% CI
Indirect effect of discrimination in medical settings on COVID-19 vaccine refusal through low confidence in the central UK government	0.94	0.32	2.77
Indirect effect of discrimination in medical settings on COVID-19 vaccine refusal through low confidence in the UK health service	0.81	0.10	6.54
Total effect of discrimination in medical settings on COVID-19 vaccine refusal (direct effect of discrimination in medical settings plus indirect effects of low confidence in government and the health system)	2.33	0.15	35.27

Note. Bold indicates $p < 0.05$. Data were weighted to the proportions of gender, age, ethnicity, country, and education obtained from the Office for National Statistics. Structural equation model adjusts for COVID-19 infection status, not being worried about catching or becoming seriously ill from COVID-19, gender, age, education level, and the presence of a long-term physical health condition.

Table S11. Sensitivity analysis: Direct effects of discrimination in service settings, confidence in government and the health system and COVID-19 vaccine refusal from the structural equation model (N = 633)

	Low confidence in the central UK government			Low confidence in the UK health system			COVID-19 vaccine refusal		
	OR	95% CI	95% CI	OR	95% CI	95% CI	OR	95% CI	95% CI
Low confidence in the central UK government (ref high)	-	-	-	-	-	-	1.11	0.19	6.57
Low confidence in the UK health service (ref high)	-	-	-	-	-	-	**8.06**	**2.22**	**29.22**
Discrimination in service settings	0.91	0.33	2.45	0.55	0.21	1.44	3.05	0.90	10.27
Model fit									
Akaike's information criteria (AIC)					2704.42				
Schwarz's Bayesian information criteria (BIC)					2860.19				
Log pseudolikelihood					-1317.21				

Note. Bold indicates $p < 0.05$. Data were weighted to the proportions of gender, age, ethnicity, country, and education obtained from the Office for National Statistics. Structural equation model adjusts for COVID-19 infection status, not being worried about catching or becoming seriously ill from COVID-19, gender, age, education level, and the presence of a long-term physical health condition.

Table S12. Sensitivity analysis: Indirect effects of discrimination in service settings, confidence in government and the health system and COVID-19 vaccine refusal from the structural equation model (N = 633)

	COVID-19 vaccine refusal		
	OR	95% CI	95% CI
Indirect effect of discrimination in service settings on COVID-19 vaccine refusal through low confidence in the central UK government	0.97	0.44	2.13
Indirect effect of discrimination in service settings on COVID-19 vaccine refusal through low confidence in the UK health service	5.09	0.68	38.32
Total effect of discrimination in service settings on COVID-19 vaccine refusal (direct effect of discrimination in service settings plus indirect effects of low confidence in government and the health system)	**35.69**	**2.93**	**434.85**

Note. Bold indicates $p < 0.05$. Data were weighted to the proportions of gender, age, ethnicity, country, and education obtained from the Office for National Statistics. Structural equation model adjusts for COVID-19 infection status, not being worried about catching or becoming seriously ill from COVID-19, gender, age, education level, and the presence of a long-term physical health condition.
